# Ultrasmall surface functionalized nanoclusters (Ni, Cu and Co) for high performance oxygen evolution catalysis

**DOI:** 10.1039/d6ra03806a

**Published:** 2026-07-06

**Authors:** Syeda Tabeer Zahra, Sajid Ullah, Hemn A. H. Barzani, Muhammad Rahman, Shaheer Jamal, Nora Mobark Farhan, Norah Alomayrah, M. S. Al-Buriahi, Ziaur Rehman, Akhtar Munir

**Affiliations:** a Department of Chemistry, Quaid-i-Azam 45320 Islamabad Pakistan akhtarmunir@qau.edu.pk zrehman@qau.edu.pk; b Department of Medical Laboratory Science, College of Health Science, Lebanese French University Erbil Iraq; c Department of Chemistry, College of Science, Princess Nourah bint Abdulrahman University P.O. Box 84428 Riyadh 11671 Saudi Arabia; d Department of Physics, College of Science, Princess Nourah bint Abdulrahman University P.O. Box 84428 Riyadh 11671 Saudi Arabia; e Department of Physics, Sakarya University Sakarya Turkey

## Abstract

Water electrolysis is an appealing approach for the production of molecular H_2_ as a sustainable and clean energy carrier. However, the intrinsic sluggish kinetics of the oxygen evolution reaction (OER) restrict its practical application. In this context, various approaches, particularly engineering of materials at the nanoscale length, offer high surface area and tunable electronic and redox properties, significantly accelerating the challenging OER process. This research primarily focuses on the development of cost-effective, thiol-stabilized transition metal ultrasmall nanoclusters (MNCs) as electrode materials, with precise control over size and surface chemistry. Herein, an optimized protocol is presented for the synthesis of transition metal nanoclusters (MNCs, M = Ni, Co and Cu) using 1-dodecanethiol and 2-phenylethanethiol as stabilizing agents. The synthesized MNCs were initially characterized using UV-visible and FT-IR spectroscopic techniques, indicating nanoclusters composed of metal-based nanoclusters stabilized by thiol ligands. Elemental composition was confirmed by EDX and XPS analysis. STEM and HRTEM revealed the uniform distribution (size ≤ 2 nm) of MNCs. The electrochemical evaluation showed that NiNCs emerged as the best electrocatalyst, exhibiting low onset potential of ∼1.46 V *vs.* RHE (*η* ≈ 230 mV), with a maximum current density of ∼125 mA cm^−2^ at 1.7 V *vs.* RHE, and showing a low Tafel slope value of 99 mV dec^−1^. The activity of Ni NCs is superior to the benchmark electrocatalysts, namely RuO_2_ (*η*_10_ ≈ 300–350 mV) and IrO_2_ (*η*_10_ ≈ 300–400 mV). Moreover, the Ni NCs also exhibit maximum exchange current density (*I*_o_ ≈ 0.9 mA cm^−2^), efficient mass activity (∼400 A g^−1^@1.7 V) and prolonged OER performance, which are indeed worth considering as attributes for further advancements in catalysis.

## Abstract 2

Development of ultrasmall metallic nanoscale materials is an appealing approach for water electrolysis for the production of molecular H_2_ as a sustainable and clean energy carrier.

## Graphical abstract

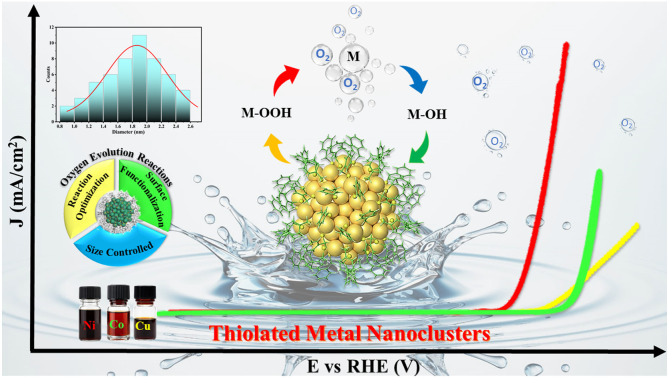

## Introduction

The persistent rise in global energy consumption has driven scientific initiatives to substantially mitigate the environmental impact of the fossil fuels around the globe.^1,2^ To reduce the reliance on carbon based fuels, it is required to develop integrated renewable energy systems (conversion and storage) to effectively meet the growing energy demands.^3–5^ Among various secondary energy carriers, hydrogen has emerged as an efficient and clean fuel owing to its high energy density (120 MJ kg^−1^) and reduced ecological footprint.^6–8^ To address the associated challenges inherent in the diverse spectrum of energy production systems, electricity driven hydrogen production from water, termed electrolysis, has garnered substantial attention.^9–11^ Electrochemical water splitting proceeds through two key half-reactions: the hydrogen evolution reaction (HER, 2H^+^ + 2e^−^ → H_2_), and the oxygen evolution reaction (OER, 2H_2_O → O_2_ + 4H^+^ + 4e^−^).^12,13^ However, the OER exhibits sluggish kinetics that ultimately result in a high overpotential, thereby representing the primary bottleneck limiting the efficiency of overall water electrolysis.^14,15^ Remarkable OER catalytic activity has been reported for noble metal based electrocatalysts, especially RuO_2_ (∼300–350 mV@10 mA cm^−2^) and IrO_2_ (∼300–400 mV@10 mA cm^−2^).^16,17^ However, their high cost and limited operational stability constrained their practical applications.^18^ The scarcity and high cost of the benchmark electrocatalysts for OER have motivated researchers to explore the development of low-cost electrode materials.^19^

In the last two decades, many efforts have been made to catalyze the water splitting process *via* both homogeneous and heterogeneous catalysis.^20^ Fe, Mn, Ir, Ru, and Co complexes have been extensively studied as homogeneous catalysts with adequate turnover frequencies (TOFs).^21,22^ Similarly, several composite materials (heterogeneous), metal chalcogenides, and members of spinel family have been thoroughly investigated. Encouragingly, some of these are extremely active, even surpassing the benchmark electrocatalysts for OER.^14–24^ Layer double hydroxides (LDHs), a class of transition metal-based electrocatalysts, have been thoroughly investigated for OER in alkaline media. LDHs are 2D materials with a brucite-like structure, shaped by the association of two metal layers with diverse oxidation states imposed by the presence of cations and anions.^25,26^ Recently, Fatemeh Razmjooei *et al.* reported heteroatoms (S, P, N, B) doped graphene-supported Fe nanocomposites for electrocatalytic water oxidation.^27,28^ To further improve the stability of the hybrid material, a series of LDH were used as support material following exfoliation of the 2D structure. Despite low conductivity of LDHs, they have been loaded onto a variety of conducting materials (*e.g.*, GO, CNTs, g-C_3_N_4_) to address the allied problems. Furthermore, they have also been integrated to porous materials (MOFs, COFs) to enhance their catalytic performance for overall water splitting.^29^

The exploration of atomic-level materials and nano-structuring signifies an emerging area of research aimed at enhancing the performance of overall electrode materials. Several methods have been adopted to stabilize the electrocatalysts by supporting them onto porous and conducting structures.^30^ The investigation of the catalytic behavior of materials in nanoscale dimensions is an exciting discovery made possible by the advancements in material synthesis.^31,32^ Moving from bulk material to the nanoscale level and then to the sub-nanometric regime, new materials have been emerged, known as nanoclusters (NCs, size ≈ 2 nm). These NCs assemblies show molecule-like behavior and excellent catalytic properties due to their high surface-to-volume ratio, high redox potential, exceptional electrical properties, and quantum confinement, thus commencing enormous interest in catalysis.^33,34^ Previous work, both experimental and theoretical, have established that engineering core size and surface chemistry of materials can further enhance their catalytic activity and selectivity.^35^ The surface functionalization of catalytic materials at sub-nanometer scale can greatly enhance the catalytic performance by facilitating rapid mass transport and charge transfer.^36^ The shorter electron transfer pathways and higher surface to volume ratio ultimately promotes faster kinetics for OER.^37^ The nano-structuring of catalytic materials may induce a cathodic shift in onset potential of oxygen evolution, which improves electrocatalytic performance in the water oxidation process.^38,39^

Herein, we report the development of thiol-based surface functionalized metal (M = Ni, Cu and Co) nanoclusters (NCs) *via* Brust-Schiffrin method.^40^ The controlled size of metal core, and uniform distribution of ultrasmall MNCs was achieved by optimizing the concentration of ligands and reaction conditions. These ultrasmall MNCs have a maximum number of active sites and optimized surface coverage, thereby improving the overall electrocatalytic performance in alkaline medium.

### Materials and method

#### Chemicals and reagents

Nickel(ii) chloride hexahydrate (NiCl_2_·6H_2_O >98%, Sigma Aldrich), cobalt(ii) chloride hexahydrate (CoCl_2_·6H_2_O, >98%, Sigma Aldrich), copper(ii) chloride (CuCl_2_, >85%, Sigma Aldrich), tetraoctylammonium bromide (TOAB, >96%, Sigma Aldrich), sodium borohydride (NaBH_4_, >98%, Alfa Aesar), potassium hydroxide (KOH, >96%, Sigma Aldrich), 2-phenylethanethiol (PET, >80%, Alfa Aesar), 1-dodecanethiol (Dodec, >80%, Sigma Aldrich). Analytical grade solvents like tetrahydrofuran (THF, >99%) and toluene (>99%) were used during reaction. Similarly, solvents like methanol, ethanol were used after distillation. Dichloromethane (DCM, >99%) was used for final dissolution and analysis.

#### Synthesis of Ni NCs

**Scheme 1:**
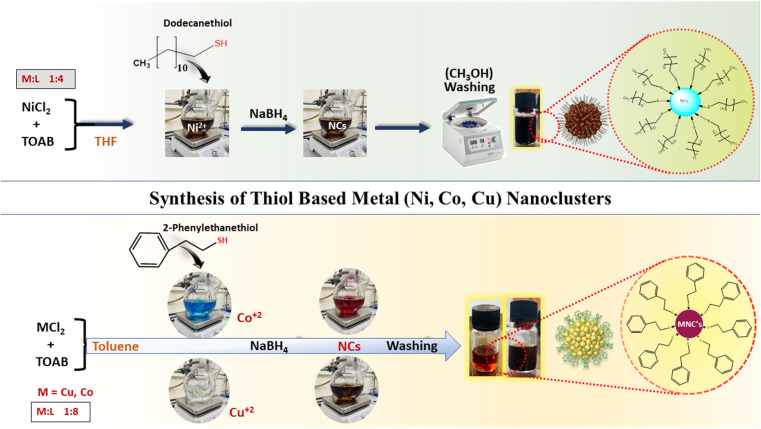
Synthetic scheme of MNCs (M = Ni, Co, Cu) nanoclusters.

The synthesis of thiol-stabilized Ni NCs was carried out using the reported Brust schiffirin method^41^ with slight modification. During synthesis, 99.84 mg (0.42 mmol) of NiCl_2_·6H_2_O and 478 mg (0.87 mmol) of tetraoctylammonium bromide (TOAB) were dissolved 100 mL of THF. After complete dissolution of metal salt, 0.52 mL (98%) of 1-dodecanethiol (2.17 mmol) as capping ligand was added. The reaction mixture was allowed to stir for 2 to 3 hours. After possible complexation, an ice-cold aqueous solution of NaBH_4_ (160 mg in 4 mL DI water) was added as a whole with fast stirring around 1000 rpm. The mixture immediately turned into a dark brown suspension. After 24 hours of continuous stirring, the mixture was concentrated using rotary evaporation followed by washing with methanol employing precipitation and dissolution method (Scheme 1). The final product was dissolved in 4–5 mL of DCM solvent.

#### Synthesis of Co NCs

Initially, the Co salt was dehydrated to improve its solubility in organic solvent. The dehydrated metal salt (CoCl_2_, 30 mg, 0.23 mmol) was added in 15 mL toluene and a small amount of TOAB, resulting in the appearance of a blue color. Upon the complete dissolution of salt, 0.1 mL (0.76 mmol) of 2-phenylethanethiol (PET) was added, turning the color from blue to dark blue. After 45 minutes of stirring, ice cold aqueous solution (80 mg of NaBH_4_ in 2 mL) was added with fast stirring. A sudden change in color was observed, indicating the reduction of metal ions. After 3 to 4 hours of continuous stirring, the mixture was washed using precipitation and dissolution process with methanol. The final product was dissolved in DCM for further analysis (Scheme 1).

#### Synthesis of Cu NCs

50 mg of CuCl_2_ (0.37 mmol) was added to a solution of tetraoctylammonium bromide (TOAB) in toluene (400 mg TOAB/75 mL toluene), and the resultant suspension was subjected to vigorous stirring. After 20 minutes of vigorous stirring, the stirring speed was reduced to a moderate to add 400 µL (3.04 mmol) of 2-phenylethanethiol. After continue stirring at moderate speed for approximately 3 hours, 140 mg of freshly prepared NaBH_4_ in ice-cold water (10 mL) was added, immediately turning the color from golden-yellow to dark brown. However, the reaction mixture was allowed to stir overnight to guarantee completion of reaction. Afterwards, separate the aqueous and organic (toluene) layers, and evaporate the toluene under vacuum to reduce the volume from 75 mL to about 5 mL. To precipitate Cu nanoclusters 45 mL of methanol was added and centrifuge the Cu nanoclusters. Repeat this process thrice to effectively remove the impurities and unreacted chemicals. The final NCs were dispersed in toluene for further use.

### Characterization and electrochemical studies of the synthesized materials

Initial characterization of metal nanoclusters was done by UV-visible spectroscopy. A Schimadzu 1800 UV-visible spectrophotometer was deployed to measure the absorption spectra of MNCs. For analysis, a dilute solution of nanoclusters was prepared in DCM. The surface functionality was investigated using Thermos Scientific-6700 FT-IR spectrophotometer covering the 4000–400 cm^−1^. The elemental composition of metal nanoclusters was determined using the JASCO V77 spectrophotometer and INCAX-Act Energy Dispersive X-ray Spectroscopy (EDX). Scanning Transmission Electron Microscopy (STEM) with an FEI NOVA-SEM-450 was used to evaluate the texture, morphology and distribution pattern of MNCs. A High-Resolution Transmission Electron Microscopy (HRTEM, JEM2100F, JEOL, 200 36 kV) was employed to examine the uniform distribution and accurate size of MNCs. XPS analysis was performed by PHI 500 Versa Probe II spectrometer (UIVAC-PHI) for the compositional study, chemical environment and purity of the samples. Gamry potentiostat 600 was used for the electrochemical evaluation in basic medium (0.1 M KOH) for OER. The electrochemical setup consists of a three-electrode system in which glassy carbon (GC, 0.07 cm^2^, working electrode), Ag/AgCl (reference electrode), and Pt wire (counter electrode) were used. Before analysis, metal nanoclusters were dissolved in 5 mL DCM and sonicated for homogeneous dispersion. The Electrochemical Impedance spectroscopy (EIS) was performed in the frequency range 1 Hz to 1 MHz maintaining the potential near the onset potential. The working electrodes were simply fabricated by drop casting and dried in an open atmosphere.

## Results and discussion

### Synthesis of materials

Ni, Co and Cu nanoclusters were synthesized using stabilizing agent like dodecanethiol and 2-phenylethanthiol under the control and optimized condition. For Ni NCs, reported procedure was followed with slight modified,^41^ using long chain dodecanethiol instead of PET to tune the hydrophobicity, core atoms and surface coverage, as these factors are worth considering in catalytic process. Notably, the preparation of Ni MNCs is straight forward and can be easily managed by controlling the reaction conditions in the presence of dodecanethiol as a capping agent. In contrast, the oxophilic nature of Cu and Co pose a significant challenge in the synthesis of their clusters. Therefore, PET was employed as a capping ligand to maintain adequate linker coverage probably facilitated by PET's aromatic ring in the colloidal form. This not only regulates the electronic environment, but also commendably manages the agglomeration and oxidation vulnerability of Co and Cu. Moreover, to get the requisite sterilizability in case of Cu NCs and Co NCs, a high ligand-to-metal ratio was used that resulted in crowding of the active center, which adversely affected their catalytic activity. Various optimization strategies have been employed to control the monodispersity and size of the metal core *i.e.*, varying the nature and ratio of ligands, TOAB, and NaBH_4_ w.r.t metal precursors and reaction conditions. Interestingly, the method presented for the synthesis of the three MNCs is highly reproducible, where the reaction time and progress can be easily monitored using UV-visible spectroscopy.

### Structural characterization

**Fig. 1:**
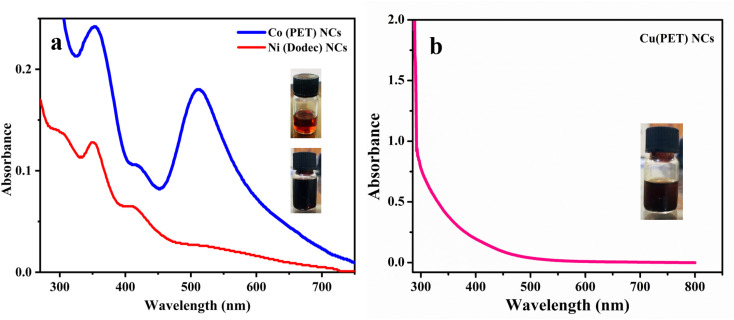
UV-visible spectra of freshly prepared nanoclusters (a) Ni (Dodec) NCs and Co(PET) NCs (b) Cu(PET) NCs.

UV-visible analysis of the synthesized materials was performed in the DCM solvent. The appearance of multiple peaks indicates the emergence of discrete energy levels, thereby suggesting the formation of metal nanoclusters. Fig. S1 shows the UV-visible spectra obtained from *in situ* sampling collected during the optimization procedure at various intervals. Prior to NaBH_4_ addition, the detected peaks at ∼430 nm and ∼500 nm may correspond to sulfur to metal charge transfer and d–d transitions, respectively, thus confirming the precursor's complexation. However, after the addition of NaBH_4_, both peaks disappeared due to the reduction of Ni^2+^ to Ni^0^. In addition to this, characteristic multiple peaks were observed (intense; ∼380 nm, ∼430 nm and broad; ∼500–550 nm) due to the possible emergence of discrete energy levels, validating the formation of nanoscale particles.^41^Fig. 1a shows the UV-vis spectra of the formed nanoclusters: Ni NCs and Co NCs, which align well with the literature.^42,43^ The comparative spectra of the Co nanoparticles and nanoclusters are illustrated in Fig. S2, which shows comparative peaks for complex formation before reduction (d–d transition; ∼420 nm, π–π* transition; ∼330 nm), for thiolate-functionalized Co NPs (broad; ∼520 nm, ∼420 nm) due to surface plasmonic resonance (SPR) and for Co NCs (∼352 nm, ∼416 nm, ∼511 nm and ∼639 nm). Fig. 1a shows the characteristic absorption peaks of Co(PET) NCs, which have also been described for other cobalt nanoclusters with different ligands.^44,45^ Optimization spectra of Co NCs (Fig. S3) clearly indicate the deviation in peaks as the ligand concentration was gradually vary. Cu NCs show no prominent peak within the 300–800 nm range (Fig. 1b) except for high intensity peak at 280 nm, which may submerge the weak d–d transition peak.^46^ FT-IR was performed to follow the interaction of thiol-based ligand with MNCs. The interaction can be signified by the disappearance of the S–H peak at ∼2500 cm^−1^ and appearance of the M-S peak at ∼500 cm^−1^.^47,48^ The FT-IR spectra of Co NCs, Cu NCs and Ni NCs (Fig. S4–S6) also show a long chain band at ∼750 cm^−1^.

**Fig. 2:**
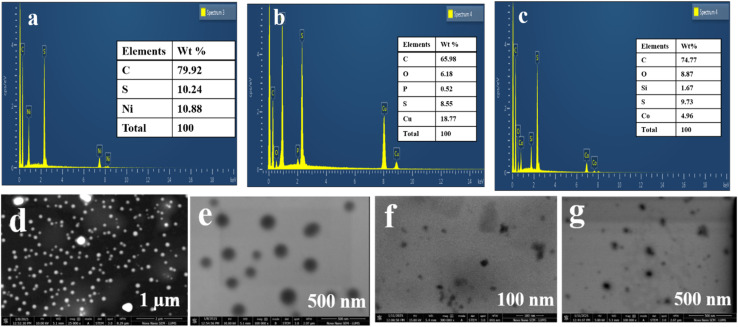
EDX along with percentage composition and STEM analysis EDX spectra of (a) Ni(Dodec) NCs (b) Cu(PET) NCs (c) Co(PET) NCs and STEM images of (d) and (e) Ni(Dodec) NCs at 1 µm and 500 nm respectively (f) Co(PET) NCs at 100 nm (g) Cu(PET) NCs at 500 nm.

**Fig. 3:**
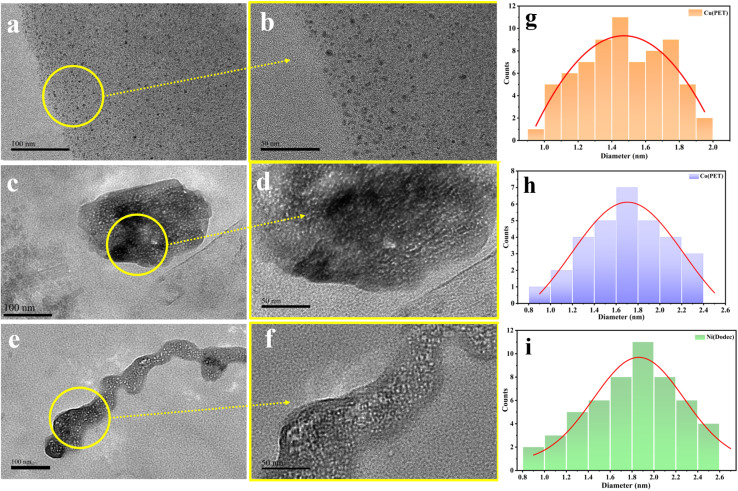
HR-TEM images are shown as (a) and (b) Cu(PET) NCs at 100 nm and 50 nm along with size distribution curve (c) and (d) HR-TEM image of Co(PET) NCs at 50 nm and 100 nm and size distribution curve of Ni(Dodec) NCs (e) and (f) HR-TEM images of Ni(Dodec) NCs at 100 nm and 50 nm. (g)–(i) Histograms of Ni, Cu and Co NCs, respectively.

EDX was employed to confirm the purity and composition of the synthesized material. EDX analysis shows the following percentage composition for MNCs: Ni NCs: Ni (79.92%), C (10.24%), and S (10.88%) (Fig. 2a), Co NCs: C (74.77%), O (8.87%) (due to formation of some oxides), S (9.73%), Co (4.96%), and a trace amount of silicon (Fig. 2c), and Cu NCs: C (65.98%), O (6.18%), S (8.55%), and Cu (18.77%) (Fig. 2b). A very small size of nanoclusters is beyond the lens of SEM and PXRD; therefore, scanning transmission electron microscopy (STEM) was employed for assessment of size of MNCs. Fig. 2d–g shows the STEM images at 1 µm and 500 nm for Ni NCs, at 100 nm for Co NCs and at 500 nm for Cu NCs. These images clearly reveal the presence of small-sized and uniformly distributed MNCs. Remaining STEM images at various magnifications for MNCs are depicted in Fig. S6–S9. Notably, STEM analysis offers a much better insight than SEM analysis regarding particle's size and their distribution. However, STEM results alone are inadequate for precise determination of particle size and uniform distribution. In this context, high resolution transmission electron microscopy (HR-TEM) was performed to get a clearer picture. The HR-TEM images confirmed the uniform distribution of particles and the formation of the nanoclusters, *i.e.*, particles size less than 3 nm. The HR-TEM images of Cu NCs (Fig. 3a and b), taken at 100 nm and 50 nm magnification, revealed that the Cu NCs appear as quantum confined dark spherical dots. The ultrasmall nature and even distribution of the Cu NCs suggest that PET, as “capping agent”, effectively shields the copper cores, precluding their aggregation into bigger particles. The TEM image, collected at magnification of 10 nm, reveals lattice fringes correspond to the crystalline core of Cu NCs and uniform particle size distribution *i.e.*, average size of 1.55 nm (calculated by taking ∼50 number of particles) (Fig. S10). Similarly, the HR-TEM images (Fig. 3c–f) unveil ultrasmall Ni NCs and Co NCs with granular morphology. The histograms (Fig. 3g–i) display the average particle size of 1.8 nm for Ni NCs and 1.6 nm for Co NCs (∼50 number of particles). The well-defined spherical morphology with uniform particle distribution can be attributed to Dodec and PET ligands, which provide a steric barrier to prevent aggregation.

**Fig. 4:**
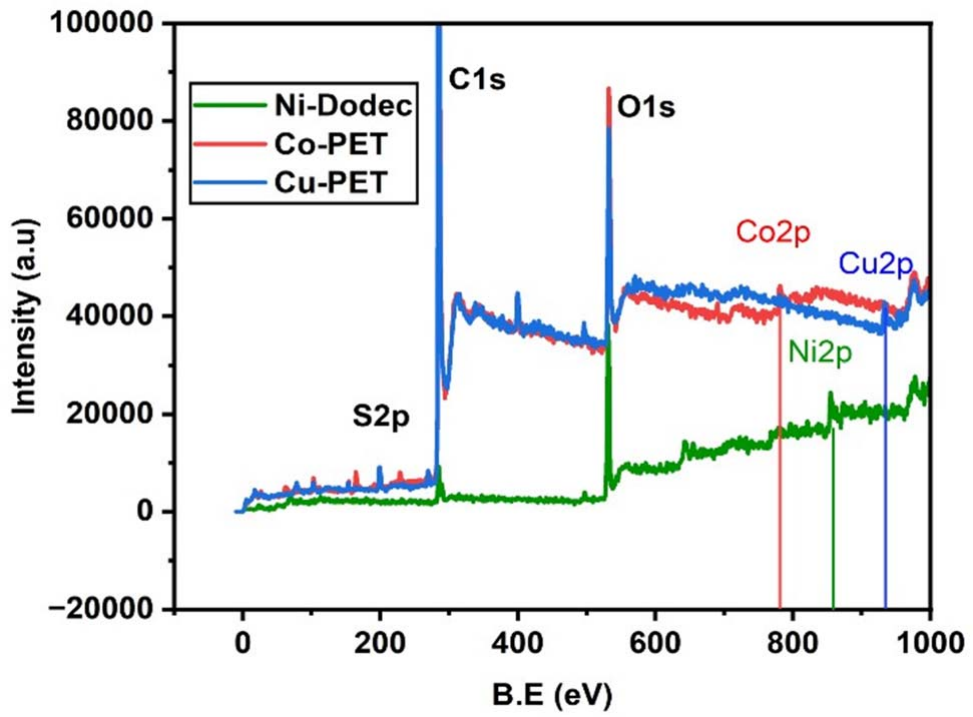
XPS survey scan of Ni, Cu, and Co nanoclusters.

**Fig. 5:**
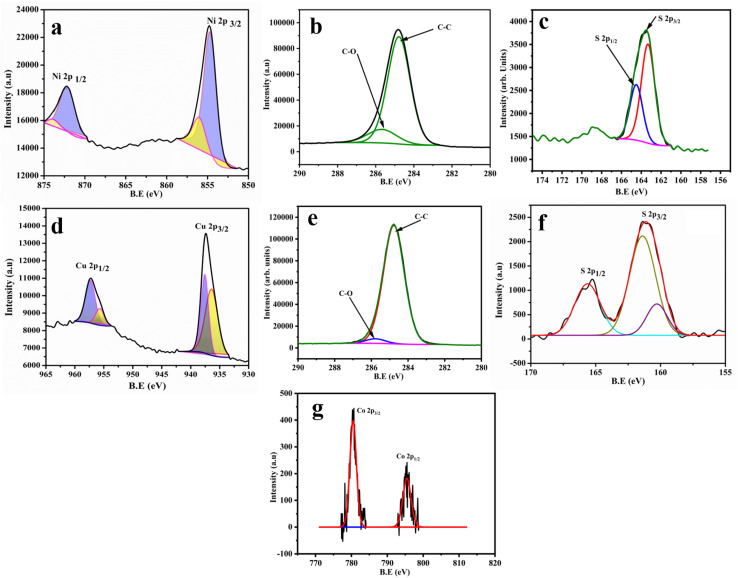
Deconvoluted XPS spectra for Ni(Dodec) NCs (a) Ni 2p (b) C 1s (c) S 2p. Core level spectra of Cu(PET) NCs (d) Cu 2p (e) C 1s (f) S 2p. Core level spectrum of metal in Co(PET) NCs (g) core level spectrum of Co 2p.

XPS is a surface sensitive technique that primarily gives information about the material's chemical composition, possible oxidation state and chemical environment.^49^ The survey scan of thiol-stabilized Ni, Cu, and Co NCs revealed the presence of the expected elements: Ni, Cu, Co, O (due to the formation of oxides) C and S (Fig. 4). The surface chemical composition and electronic states of the Ni NCs were validated by core-level XPS analysis, which confirms the formation of thiol-functionalized stable metallic Ni core. The Ni 2p spectrum (Fig. 5a) exhibits characteristic spin–orbit splitting, with the Ni 2p_3/2_ and 2p_1/2_ peaks positioned at ∼853.8 eV and 871.2 eV, respectively. These binding energies indicate the presence of metallic Ni^0^ core, with slight electronic changes due to its surface functionalization with thiol linkers. Additionally, a satellite peak at about 862 eV indicates the occurrence of Ni^2+^ species, likely originating from surface oxidation of Ni NCs upon exposure to air. The atomic ratio of Ni^0^ : Ni^2+^, calculated from the integrated peak areas with the appropriate sensitivity factor, is ∼4 : 1, confirming the predominance of the metallic state. However, the minor presence of oxides can be attributed to surface oxidation. Detailed calculations of the foregoing ratio are given in the Section S1.1. The presence of oxygen further confirms the foregoing fact (Fig. 5b). Complementary analysis of the C 1s region (Fig. 5b) depicts a dominant peak at ∼284.8 eV, which corresponds to the sp^3^ hybridized carbons of the alkyl chain of the dodecane. This is accompanied by a minor shoulder at ∼286.2 eV, which can be attributed to adventitious C–O species. The effective ligand grafting is further validated by the S 2p spectrum (Fig. 5c), which reveals a prominent S 2p_3/2_ peak at ∼162.4 eV, a classic signature of Ni–S bond formation. Moreover, the absence of signals in the 167–169 eV region confirms the sample is free of oxidized sulfur species. The foregoing results collectively confirm the stable Ni NCs, which is characterized by a metallic core encapsulated within dodecanethiol ligands.^50^ The Cu 2p core-level spectrum exhibits two primary peaks at ∼932.5 eV and 952.3 eV, assignable to the Cu 2p_3/2_ and Cu 2p_1/2_, respectively, which correspond to the different oxidation states of copper *i.e.* Cu^2+,0,1+^. The primary peaks at ∼932.5 eV and ∼952.3 eV due to the Cu 2p_3/2_ and Cu 2p_1/2_ can be assigned to the metallic state. Similarly, peaks appearing at higher binding energies can be ascribed to Cu d^9^ system *i.e.* Cu^1+/2+^2p_3/2_ and Cu ^1+/2+^2p_1/2_, respectively^51^ (Fig. 5d). The peak area for Cu 2p spectra was also evaluated, substantiating a Cu^0^ : Cu^2+^ ratio of 1.5 : 1, which confirms the presence of metallic core alongside surface oxidation. Despite the relative air sensitivity and redox trend of the transition metals; controlling their surface oxidation is extremely challenging, especially in the sub nanometric regime. Therefore, surface oxidation was noted in all metals examined. The core-level C 1s spectrum exhibits a main peak at ∼284.8 eV, representing the C–C/C–H bonds of the PET ligand framework. Additionally, a weak shoulder at a higher binding energy of ∼286.2 eV corresponds to C–S bonding (Fig. 5e). This is further corroborated by the S 2p peak (Fig. 5f), which displays a well-resolved doublet with the S 2p_3/2_ peak centered at ∼162.1 eV. The spotted shift in binding energy toward lower value, compared to free thiols at ∼164 eV, provides decisive evidence of the successful chemisorption of PET ligands onto the metal surface. The existence of stable Cu–S–thiolate interaction avoid particle agglomeration and maintain chemical integrity of the NCs core. The Co 2p core-level spectrum (Fig. 5g) presents two main peaks with distinct binding energies at ∼780.2 eV for Co 2p_3/2_ and ∼796.3 eV for Co 2p_1/2_, confirming +2 oxidation state of cobalt.^52^ The coexistence of multiple oxidation states in metal nanoclusters causes a synergistic effect that enhances the catalytic performance. The electron rich metallic core facilitates efficient charge transfer, while surface metal centers in higher oxidation states provide sites for the adsorption and activation of water molecules. This core–shell electronic interplay optimizes the electron transport and surface reaction kinetics, thereby improving the overall catalytic efficiency.^33^

### Electrochemical evaluation

The synthesized NCs (∼15 mg) were dispersed in 5–6 mL DCM. For the electrochemical evaluation, the dispersed nanoclusters were sonicated to gain homogenization and to minimize the possible aggregation. The glassy carbon electrode was polished sequentially with alumina slurry on micro cloth pads, and then dried at room temperature. A sample of ∼10 µL was taken and drop casted on a clean glassy carbon electrode (GCE). In this way, the mass loaded on GCE (3 mm diameter, 0.07 cm^2^ geometric area) was around 0.2 mg/10 µL. The drop-casted GCE was dried at room temperature for 1 hour. Based on the initial ink concentration, the resulting loaded catalyst was calculated to be 0.2 mg (200 µg). For reproducibility, all the electrodes were prepared in duplicate and loading consistency was verified by measuring the difference in electrode masses before and after loading using a microbalance. For GCE (0.07 cm^2^, 3 mm diameter), the mass loading was calculated to be 2.83 mg cm^−2^. All the electrochemical measurements were taken in a 0.1 M KOH electrolyte.

**Fig. 6:**
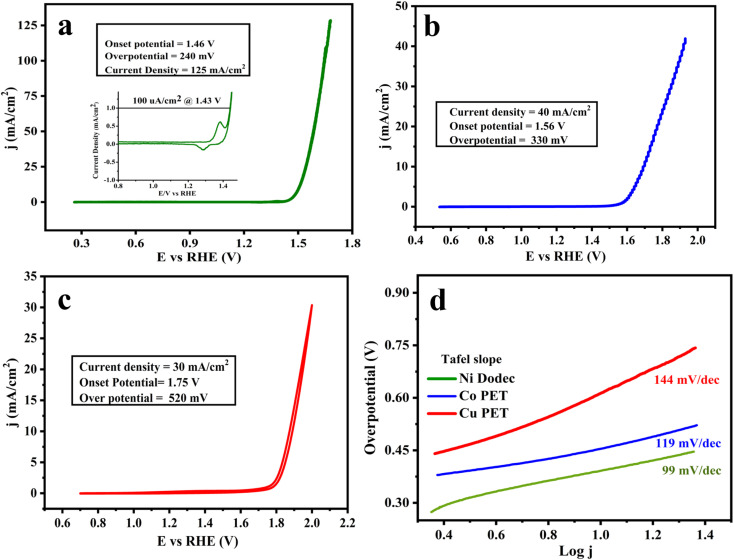
Electrocatalytic performance of the synthesized NCs. Cyclic voltammograms (a) Ni(Dodec) NCs (b) Co(PET) NCs (c) Cu(PET) NCs and (d) plots of Tafel slope.

Cyclic voltammograms of all the synthesized materials for OER are shown in Fig. 6a–d. For Ni NCs, the appearance of the redox peak of Ni^2+^/Ni^3+^ shows the electrochemical accessibility of Ni as a redox active center. A low onset potential of 1.46 V *vs.* RHE (*η* = 230 mV calculated from eqn S(2)), a maximum current density of 125 mA cm^−2^ and a Tafel slope of 99 mV dec^−1^ endorse the good kinetic viability of Ni NCs for OER.^53^ Similarly, Co NCs shows the onset potential of 1.56 V *vs.* RHE (*η* = 330 mV), a maximum current density of 40 mA cm^−2^ and a Tafel slope of 119 mV dec^−1^, which illustrates its sluggish kinetics than Ni NCs. The Cu NCs was observed to be the least active for OER *i.e.* the onset potential of 1.75 V *vs.* RHE (*η* = 520 mV), a current density 30 mA cm^−2^ and a Tafel slope 144 mV dec^−1^. In nutshell, OER performance of MNCs varies in the sequence: Ni NCs > Co NCs > Cu NCs.

**Fig. 7:**
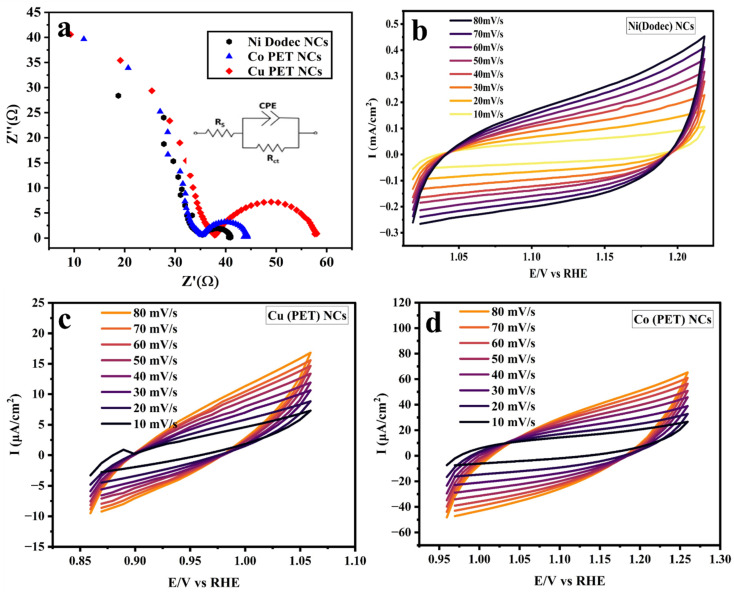
(a) EIS analysis (Nyquist plot) of NCs. Cyclic voltammograms of the synthesized NCs collected in the non-faradic region at various scan rate for the calculation of *C*_dl_ (b) Ni(Dodec) NCs (c) Cu(PET) NCs (d) Co(PET) NCs.

EIS analysis was performed within a frequency range of 0.1 to 1 MHz. Nyquist plots (Fig. 7a) were used to calculate the solution resistance (*R*_s_) and charge-transfer (*R*_ct_) resistance. Notably, *R*_s_ was taken from the high frequency region of the semicircle, while *R*_ct_ was derived from low frequency region. The *R*_ct_ values for MNCs varies in the sequence 7 Ω (Ni NCs), < 10 Ω < (Co NCs) < 20 Ω (Cu NCs). Thus, Ni NCs exhibit low charge transfer resistance and better OER activity than that of Co NCs and Cu NCs. The double layer capacitance (*C*_dl_) was calculated by analyzing the voltammograms in the non-faradic region at different scan rates (10–100 mV s^−1^). Fig. 7b–d shows the cyclic voltammograms taken in non-faradic region within the potential window of 0–1.25 V for Ni NCs, 0–1.30 V for Co NCs and 0–1.10 V for Cu NCs Vs RHE. *C*_dl_ was calculated as 22 mF cm^−2^ (Ni NCs), 47.62 µF cm^−2^ (Co NCs), and 47.60 µF cm^−2^ (Cu NCs) (Fig. S11–S13). The electrochemical active surface area (ECSA) was calculated using the specific capacitance of the electrode *i.e.* 0.04 mF. The higher *C*_dl_ value of Ni NCs gives a higher ECSA of 550 cm^2^ than that of Co NCs (1.191 cm^2^) and Cu NCs (1.190 cm^2^) (eqn S(3)).

**Fig. 8:**
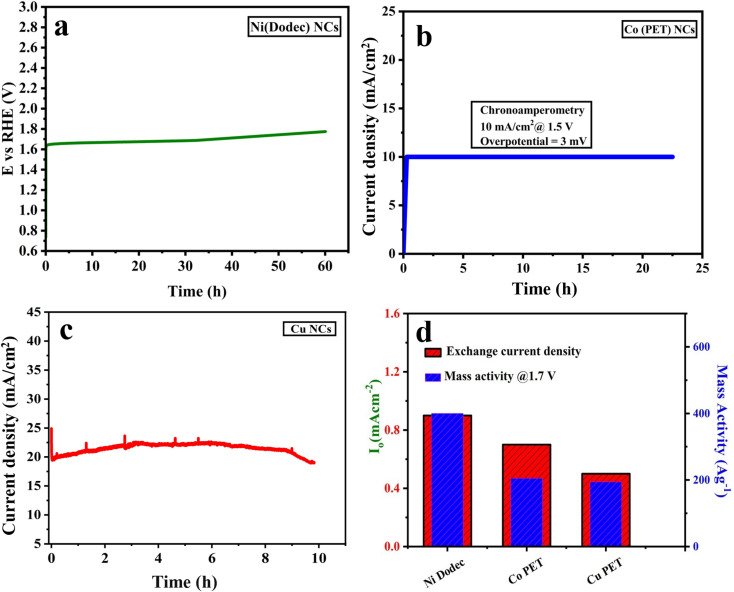
Stability test of the synthesized NCs (a) Ni(Dodec) NCs at 1.6 V (b) Co(PET) NCs at 10 mA cm^−2^ (c) Cu(PET) at 20 mA cm^−2^ (d) Comparative study of synthesized nanoclusters on the basis of mass activity and exchange current density.

To evaluate the commercial applicability of the electrocatalysts towards the OER, the electrochemical stability was assessed through chronopotentiometry as well as chronoamperometry in basic condition (Fig. 8a–c). All the synthesized electrocatalysts reveals the distinct durability trends compared to many already reported electrocatalyst (Table 1) that reflects their synthetic and intrinsic catalytic robustness. Ni NCs exhibit the highest operational stability maintaining steady performance for 60 h at the constant current density of 10 mA cm^−2^ without noticeable degradation. In contrast Co NCs sustained stable operation for 25 h only at a fixed applied potential of 1.5 V. This suggests gradual deactivation and active site passivation. Cu NCs demonstrate comparatively limited stability for 10 hours only at 1.6 V before significant performance decay, indicating a weaker structural integrity and fast degradation kinetics under the provided electrochemical condition.

**Table 1:** Comparison of MNCs with reported metal based electrocatalysts

Metal based electrocatalyst	*E*_onset_ *vs.* RHE)	*η*_10_ (mV)	Tafel slope	Durability	References
Ni_6_(PET)_12_	1.54	470	69	3 h	41
AgO_*x*_-NP	1.53	350	40	>25 h	54
Colloidal NiO NPs	—	320	40	—	55
Ni_4_(PET)_8_	1.51	330	38	>25 h	41
NiO@TiO_2_	—	320	52	10 h	56
Pd_6_(SC_12_H_26_)_12_	1.40	740	230	500 cycles	57
Au_25_/CoSe_2_	1.59	430	—	1000 cycles	58
Cu_14_ NCs	1.53	306	—	30 h	59
Cu_13_ NCs	1.61	382	—	30 h	59
Ni NC@CN	1.55	320	—	—	60
CuAu_24_(SR)_18_	—	340	90	—	61
Ni_5_(SR)_10_	1.45	370	95	—	62
Au_15_Ag_23_/NiFe-LDH		250	—	50 h	63
Ni_6_(PET)_6_	1.71	470	62	3 h	64
Ni NCs	1.45	240	99	60 h	**This work**
Co NCs	1.56	330	119	25 h	**This work**
Cu NCs	1.75	550	144	9 h	**This work**

The comparison of exchange current densities and mass activities of all the MNCs *i.e.*, Ni NCs, Co NCs and Cu NCs are shown in Fig. 8(d). The Ni NCs exhibit maximum exchange current density of ∼0.9 mA cm^−2^ followed by the efficient kinetics with low activation barrier and higher mass activity around ∼400 A g^−1^. The appreciable electrocatalytic performance of Ni NCs can be assigned to the controlled metal to ligand ratio, which in turn ensure the maximum exposure of active sites for catalysis (higher ECSA) and better OER performance as compared to Cu NCs and Co NCs. In the latter case, increased amount of metal to ligand was adopted to control size and prevent oxide formation, that buried the active sites required for OER. Additionally, inherent capacity of Ni *i.e.* its intrinsic electronic structure, redox flexibility, and ability to form highly active oxyhydroxide phases are better aligned with OER requirements.^65^

Taking into account the electrocatalytic performance, the enhanced OER activity of the Ni(Dodec) ultrasmall NCs may arise from the surface ligands and a synergistic interplay between the metallic Ni^0^ and the surface Ni^2+^ valence states. The metallic Ni^0^ core serves as an electron reservoir, facilitating rapid charge transfer from electrode to the catalytic active sites, thus minimizing overpotential losses. Concurrently, the surface Ni^2+^ species act as the primary active centers for the adsorption of OH^−^ ions and formation of key OER intermediates (OH, O and OOH*). The synergistic electronic coupling between Ni^0^ core and the surface layer covered with hydrophobic linker optimizes the binding energy of oxygenated intermediates according to the Sabatier principle. This phenomenon could lower the activation energy for the O–O bond formation, which is the rate determining step of the OER.^66,67^

Comparison of OER activity of MNCs with selected reported catalysts (Table 1) reveals that a cost-effective Ni NMCs is a highly efficient and stable catalyst for OER.

## Conclusion

In summary, the article presents efficient synthetic methods for metal nanoclusters, which is governed by the nature of the metal precursor, reaction conditions, solvent environment, and choice of the organic linker. These modulations collectively enable precise control over cluster formation within the sub-nanometric regime. The UV-vis spectroscopic analysis revealed multiple absorption features, particularly for Ni NCs and Co NCs, thus confirming the possible emergence of discrete electronic states characteristics of ultrasmall nanoscale systems. The formation of metal sulfur (M–S) bond was designated by FT-IR, as evidenced by the appearance of the characteristic band at 509 cm^−1^ and the concomitant disappearance of the S–H stretching vibration. The EDX analysis confirmed the anticipated elemental compositions, indicating high purity of the MNCs. Morphological investigations using STEM and HRTEM demonstrated a uniform and well-dispersed distribution of NCs, thus highlighting the robustness and reproducibility of the synthetic strategy. Moreover, the XPS analysis has confirmed the coexistence of multiple oxidation states within metal nanoclusters, which improves the catalytic performance. The electron rich metallic core enables efficient charge transfer, while the oxidized metals on surface serve as sites for the adsorption and activation of water molecules. Electrochemical studies have revealed notable variations in performance among Ni, Co, and Cu nanoclusters. Interestingly, Ni-based nanoclusters exhibited the lowest overpotential (240 mV), the smallest Tafel slope (99 mV dec^−1^), and commendable operational stability (60 h), surpassing their Co and Cu analogues. The perceived variations in catalytic behavior can be attributed to the intrinsic electronic structure of the metal centers, atomic arrangement within the cluster core, and surface coordination chemistry. In the future, the catalytic performance of MNCs can be improved by the rational design of bimetallic nanoclusters. This tactic is anticipated to foster synergy in the electronic and geometric interactions between two distinct metal species, which is likely to modulate the d-band center, enhance charge transfer, and create new active sites with superior intrinsic activity compared to their monometallic counterparts. Furthermore, the immobilization of both monometallic and bimetallic nanoclusters on suitable conductive supports, such as graphene oxide (GO), carbon nanotubes (CNTs), or other carbon-based frameworks, may be useful to enhance electrical conductivity, prevent agglomeration, improve mass transport, and ensure better mechanical and electrochemical stability during long-term operation.

## Conflicts of interest

The authors declare no conflict of interest.

## Supplementary Material

RA-OLF-D6RA03806A-s001

## Data Availability

The authors confirm that the data supporting the findings of this study are available within the article and supplementary information (SI) file. Furthermore all related data is available from the corresponding author on request. Supplementary information is available. See DOI: https://doi.org/10.1039/d6ra03806a.
