# Supplementary material for: Ultrasmall surface functionalized nanoclusters (Ni, Cu and Co) for high performance oxygen evolution catalysis
Source: RSC Adv. 2026 Jul 6;16(35):36894–906. doi: 10.1039/d6ra03806a (PMC13334380; doi:10.1039/d6ra03806a)
Supplement: RA-016-D6RA03806A-s001 [file RA-016-D6RA03806A-s001.pdf]

Supplementary Information (SI) for RSC Advances.
This journal is © The Royal Society of Chemistry 2026

Supplementary file

Ultrasmall surface functionalized nanoclusters (Ni, Cu and Co) for high performance oxygen evolution catalysis

Syeda Tabeer Zahra[1], Sajid Ullah[1], Hemn A. H. Barzani[2], Muhammad Rahman[1], Shaheer Jamal[1], Nora Mobark Farhan[3], Norah Alomayrah[4], M. S. Al-Buriahi[5], Ziaur Rehman*[1], Akhtar Munir[1*]

[1]Department of Chemistry, Quaid-i-Azam, 45320, Islamabad, Pakistan

[2]Department of Medical Laboratory Science, College of Health Science, Lebanese French University, Erbil, Iraq

[3]Department of Chemistry, College of Science, Princess Nourah bint Abdulrahman University, P.O. Box 84428, Riyadh 11671, Saudi Arabia

[4]Department of Physics, College of science, Princess Nourah bint Abdulrahman University, P.O. Box 84428, Riyadh 11671, Saudi Arabia

[5]Department of Physics, Sakarya University, Sakarya, Turkey

*Corresponding Author Email:
akhtarmunir@qau.edu.pk (A. Munir)
zrehman@qau.edu.pk (Z. Rehman)

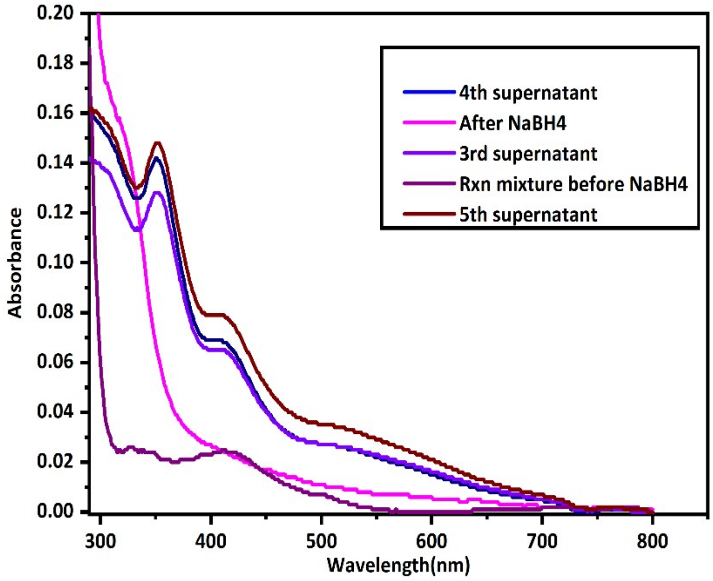

Figure S1. UV-Visible spectra for the optimization of Ni(Dodec) NCs.

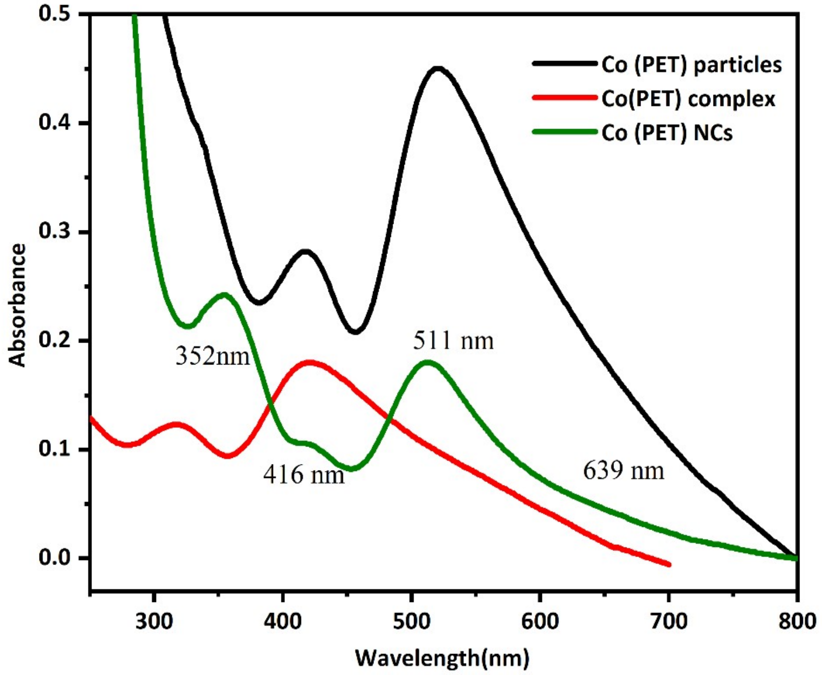

Figure S2. Comparative UV-Visible absorption data of Co(PET) nanoclusters, nanoparticles and complex.

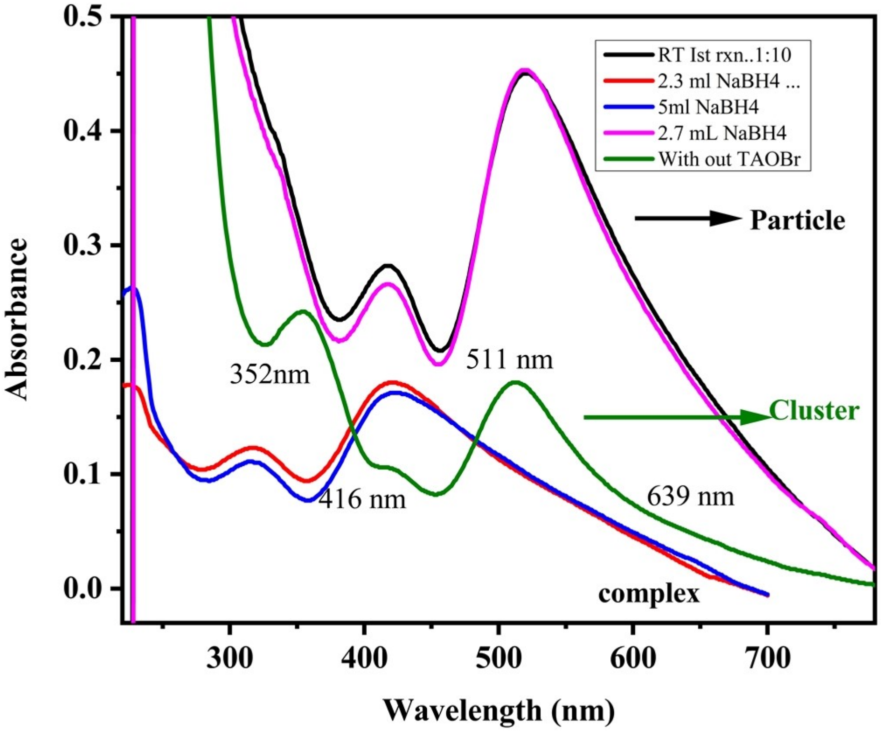

Figure. S3. UV-Visible spectra for the optimization of Co(PET) NCs

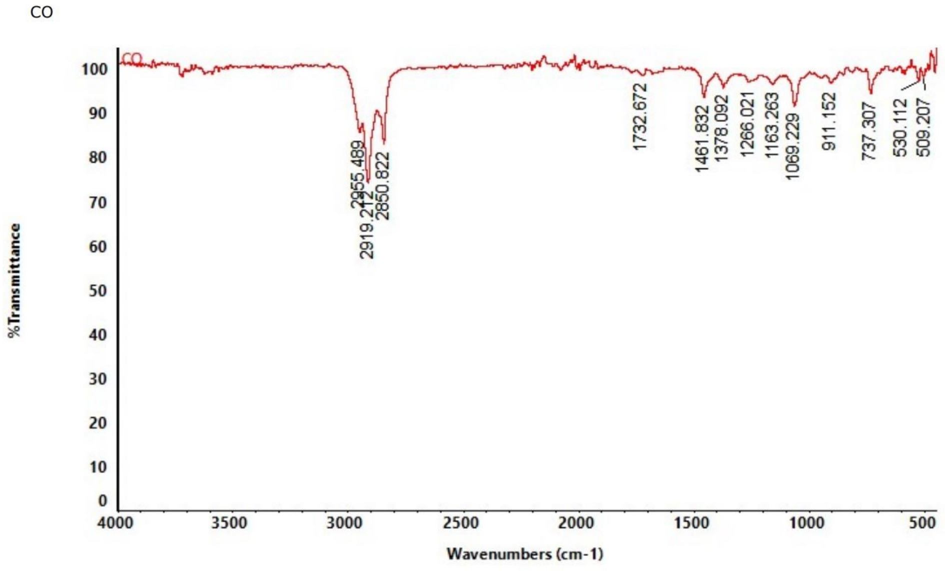

Figure S4. FT-IR spectrum of Co(PET) nanoclusters

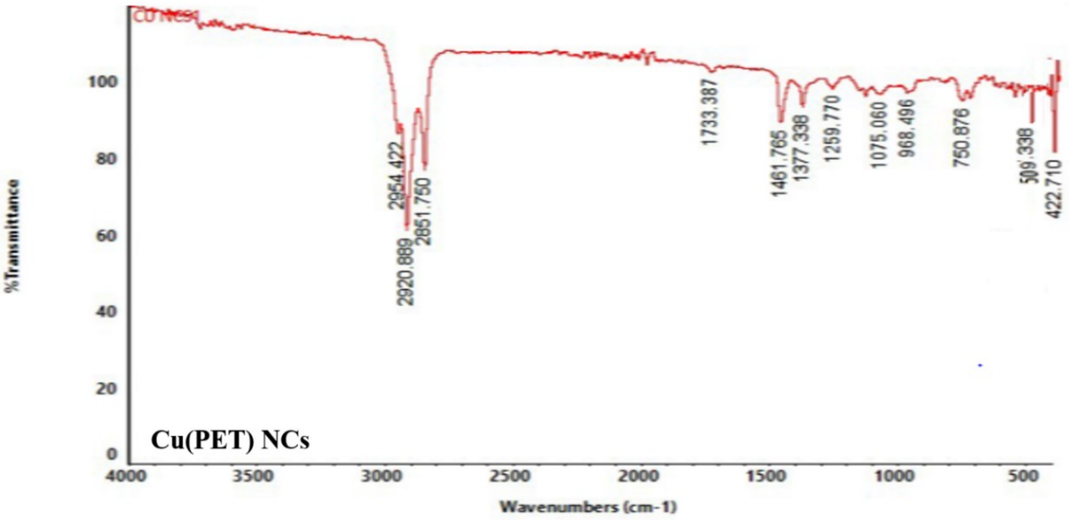

Figure S5. FT-IR spectrum of Cu(PET) nanoclusters

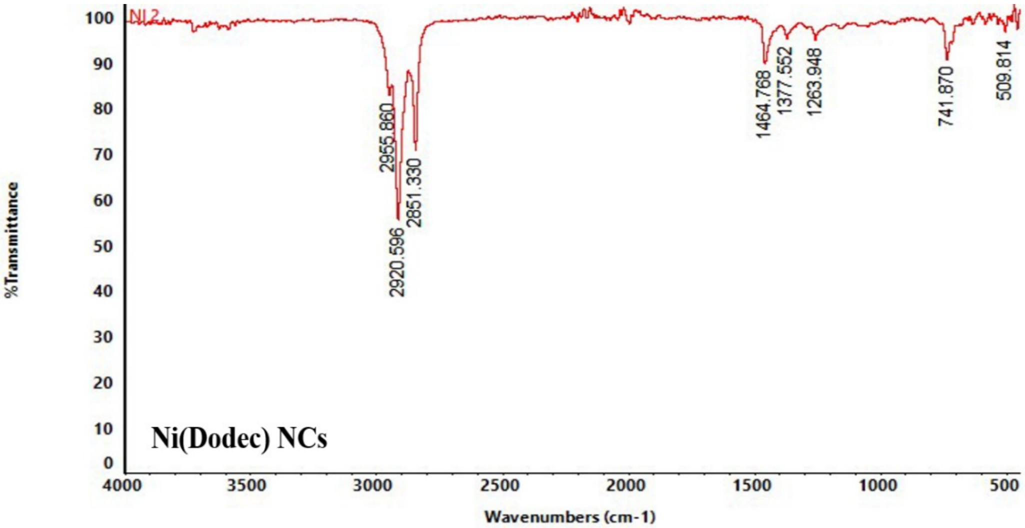

Figure S6. FT-IR spectrum of Ni(Dodec) nanoclusters

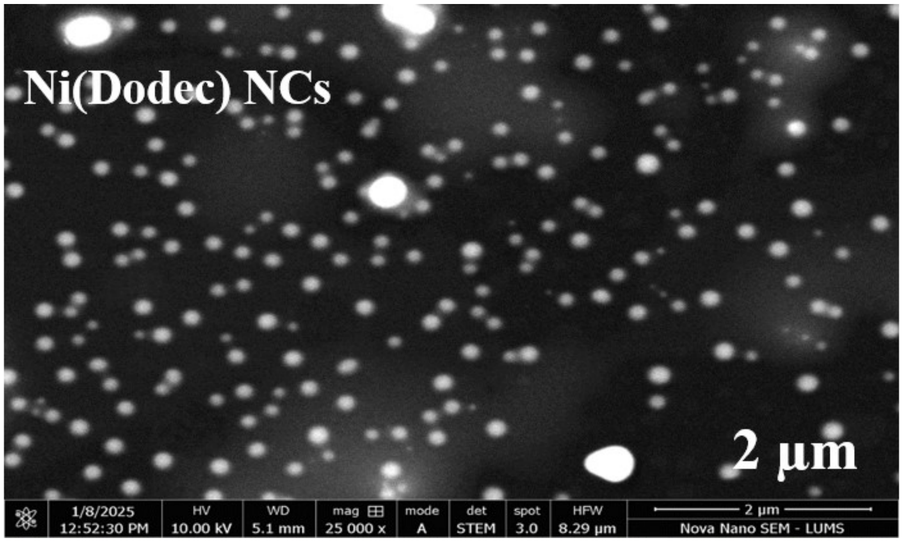

Figure S6. STEM image of Ni(Dodec) NCs at 2 µm.

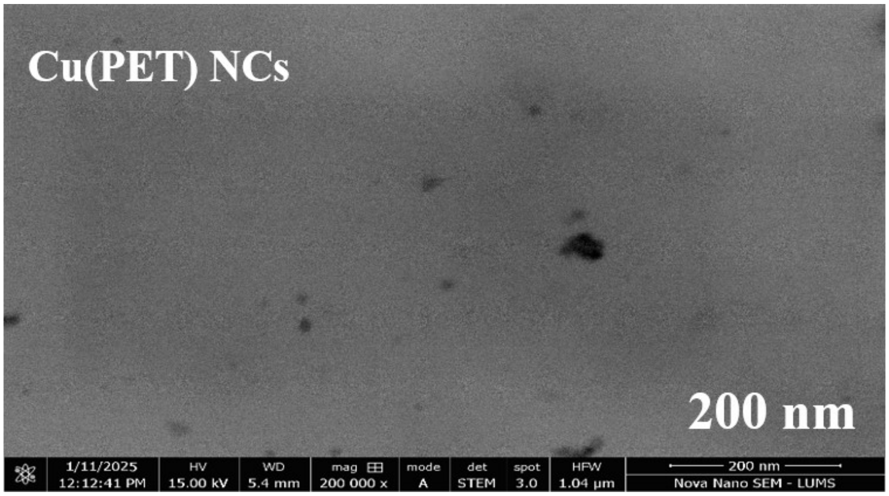

Figure S7. STEM image of Cu(PET) NCs at 200 nm

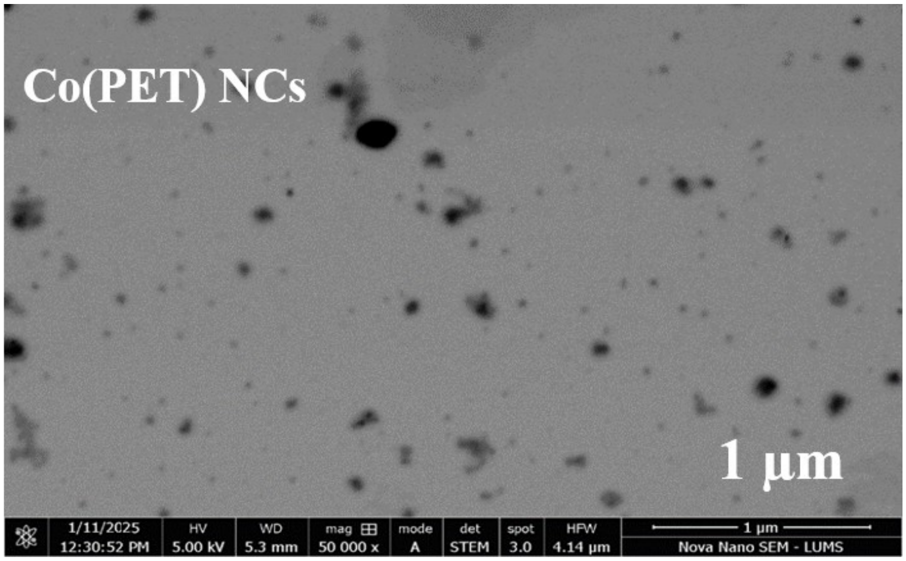

Figure S8. STEM image of Co(PET) NCs at 1μm.

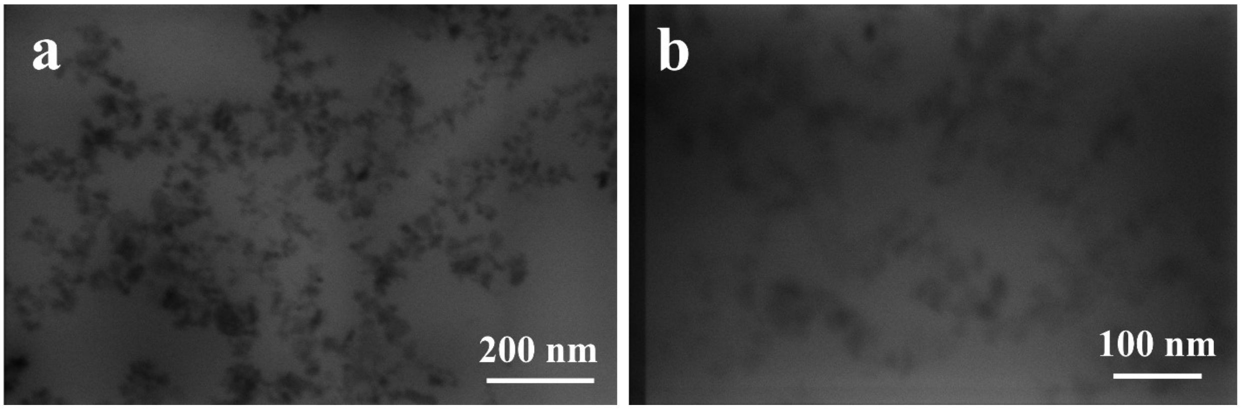

Figure S9. STEM images of Co(PET) NCs at (a)100nm (b) 200nm

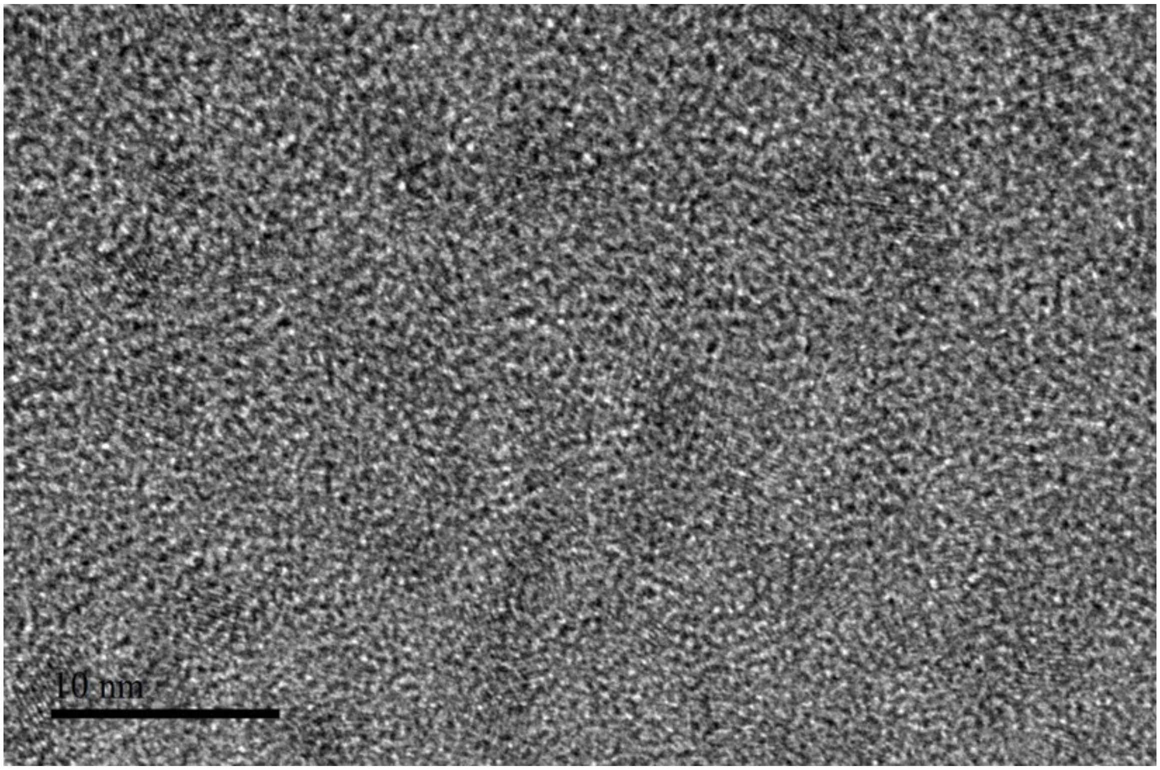

Figure S10. HR-TEM images of Cu(PET) NCs at 10 nm.

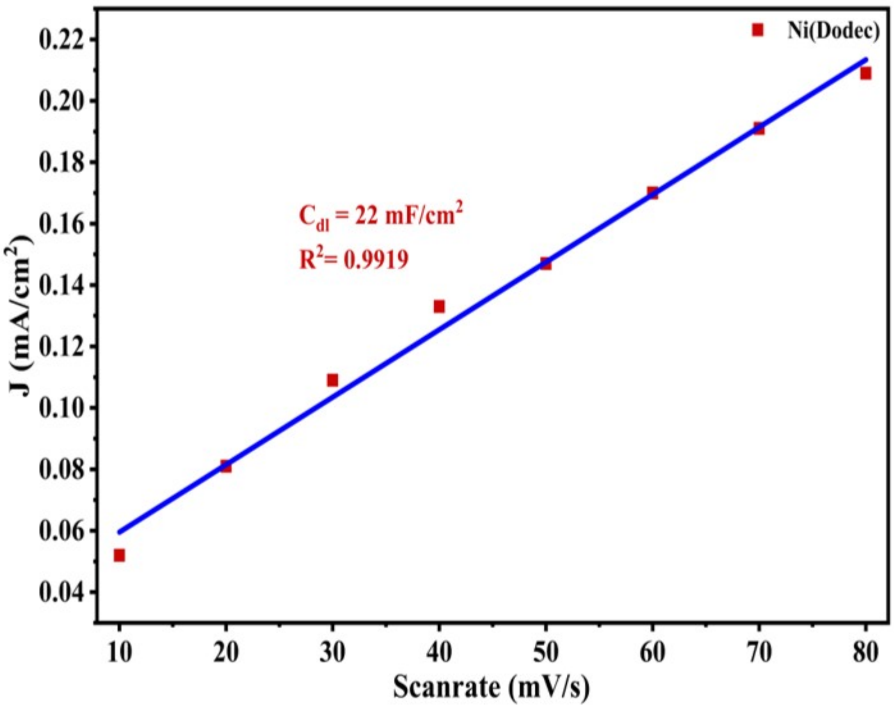

Figure S11. C_{dl} slope of Ni(Dodec) NCs

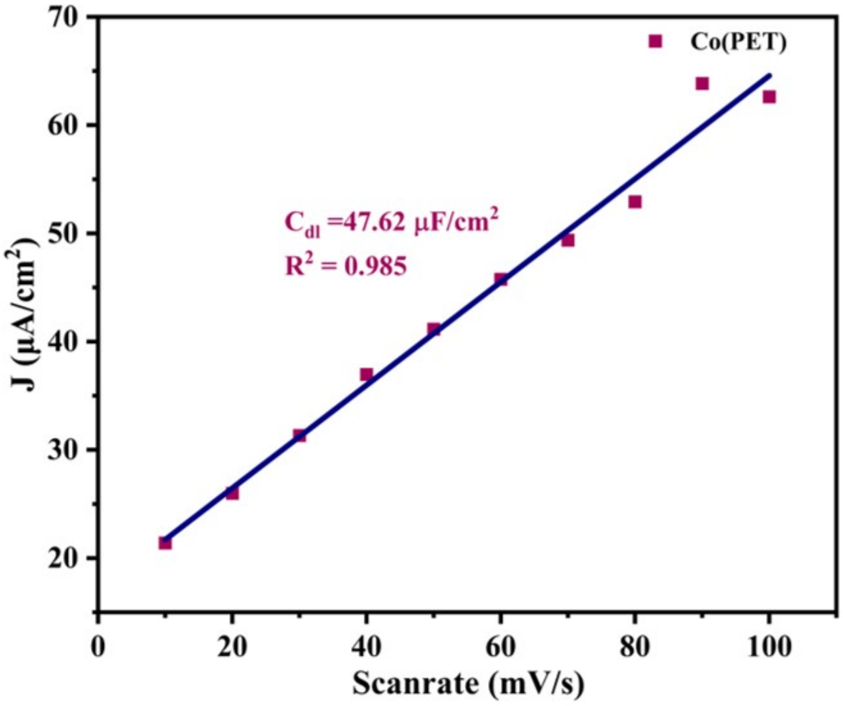

Figure S12. C_{dl} slope of Co(PET) NCs

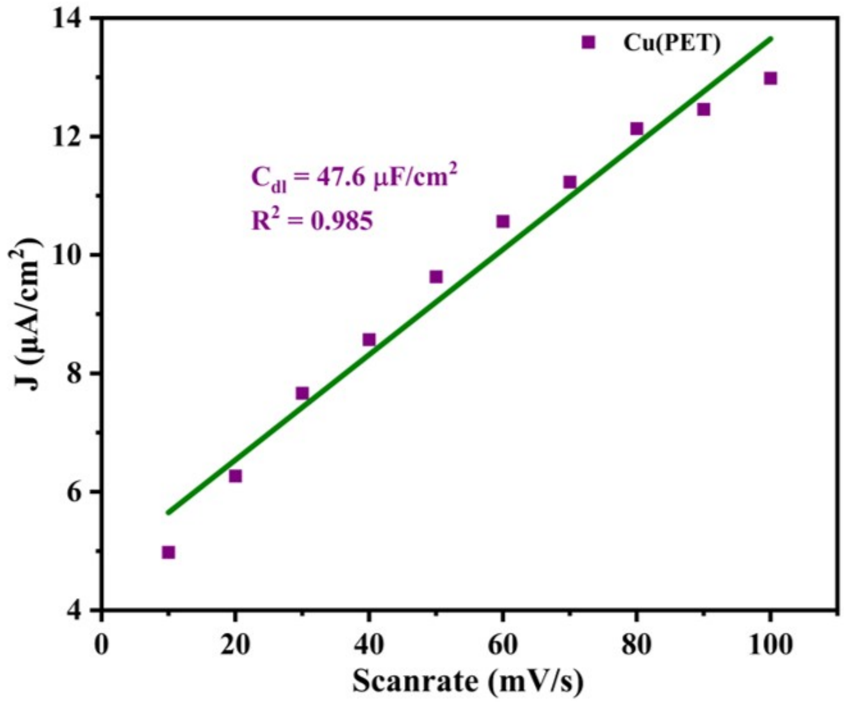

Figure S13. C_{dl} slope of Cu(PET) NCs

S 1.1 XPS peak area Ratio:

For Ni NCs (peak area)

2p1/2	2p3/2
Ni^0:Ni^{+2}	Ni^0:Ni^{+2}
44416:12166	80549:33475
**4:1**	**2.42:1** (a rough estimated ratio)

Actual and Thersitical loading of the catalyst on GCE:

Actual Loading = Weight of electrode after deposition - Weight of electrode before deposition

$$L = 5.6610\ g - 5.6608\ g$$

$$L = 0.0002\ g\ =\ 0.2\ mg$$

As GCE surface area is 0.07 cm^2, thus the catalyst mass loading is **2.83 mg/cm^2.**

Nominal Loading Calculation:

$$L = \frac{C\ \times V\ \times\ F_{active}\ \times 1000}{A} \qquad \textbf{Eq.S-1}$$

L = Catalyst Loading (μg cm^{-2})

C = Concentration of Metal Nanoclusters in the catalyst ink (mg mL^{-1})

V = Volume of ink deposited onto the electrode (μL)

F_{active} = Mass Fraction of active metal nanoclusters in the dried catalyst layer (dimensionless; $f_{active} - f_{binder}$) [No use of F_{active} as we have not use any binder]

A = Geometric surface area of the working electrode (cm^2)

1000 = Conversion factor from μg to mg (since 1 mg = 1000 μg and C is in mg mL^{-1}, V in μL, the factor of 1000 ensures the final unit is μg cm^{-2})

$$L = \frac{0.02 \times 10 \times 1000}{0.07}$$

$$L = \frac{200}{0.07}$$

$$L = 2857.14\ \mu gcm^{-2}$$

$$L = 2.86\ mgcm^{-2}$$

Actual and theoretical calculation for mass loading of catalyst on GCE is almost same.

Overpotential η

$$\eta = E_{RHE} - 1.23V \qquad \text{(Eq.S-2)}$$

For Ni (Dodec) NCs

η = 1.47 – 1.23 V

= 0.240 V

η = 240 mV

For Co (PET) NCs

η = 1.56 – 1.23 V

= 0.330 V

η = 330 mV

For Cu (PET) NCs

η = 1.75 – 1.23 V

= 0.520 V

η = 520 mV

Electrochemical active Surface Area (ECSA)

$$ECSA = C_{dl}/C_s \qquad \text{(Eq.S-3)}$$

For Ni (Dodec) NCs

ECSA= 22mF/cm^2 / 0.04mF

= $550 cm^2$

For Co (PET) NCs

C_{dl} = 47.62 μF/cm^2

=0.04762 mF/cm^2

ECSA =1.191 cm^2

For Cu (PET) NCs

Cdl = 47.60μF/cm^2

= 0.04760 mF/cm^2

ECSA = 1.19 cm^2
